# Circadian adaptations to meal timing: neuroendocrine mechanisms

**DOI:** 10.3389/fnins.2013.00185

**Published:** 2013-10-14

**Authors:** Danica F. Patton, Ralph E. Mistlberger

**Affiliations:** Department of Psychology, Simon Fraser UniversityBurnaby, BC, Canada

**Keywords:** circadian, food entrainment, ghrelin, leptin, melanocortin, corticosterone, orexin, insulin

## Abstract

Circadian rhythms of behavior and physiology are generated by central and peripheral circadian oscillators entrained by periodic environmental or physiological stimuli. A master circadian pacemaker in the hypothalamic suprachiasmatic nucleus (SCN) is directly entrained by daily light-dark (LD) cycles, and coordinates the timing of other oscillators by direct and indirect neural, hormonal and behavioral outputs. The daily rhythm of food intake provides stimuli that entrain most peripheral and central oscillators, some of which can drive a daily rhythm of food anticipatory activity if food is restricted to one daily mealtime. The location of food-entrainable oscillators (FEOs) that drive food anticipatory rhythms, and the food-related stimuli that entrain these oscillators, remain to be clarified. Here, we critically examine the role of peripheral metabolic hormones as potential internal entrainment stimuli or outputs for FEOs controlling food anticipatory rhythms in rats and mice. Hormones for which data are available include corticosterone, ghrelin, leptin, insulin, glucagon, and glucagon-like peptide 1. All of these hormones exhibit daily rhythms of synthesis and secretion that are synchronized by meal timing. There is some evidence that ghrelin and leptin modulate the expression of food anticipatory rhythms, but none of the hormones examined so far are necessary for entrainment. Ghrelin and leptin likely modulate food-entrained rhythms by actions in hypothalamic circuits utilizing melanocortin and orexin signaling, although again food-entrained behavioral rhythms can persist in lesion and gene knockout models in which these systems are disabled. Actions of these hormones on circadian oscillators in central reward circuits remain to be evaluated. Food-entrained activity rhythms are likely mediated by a distributed system of circadian oscillators sensitive to multiple feeding related inputs. Metabolic hormones appear to play a modulatory role within this system.

## Introduction

**Figure 1:**
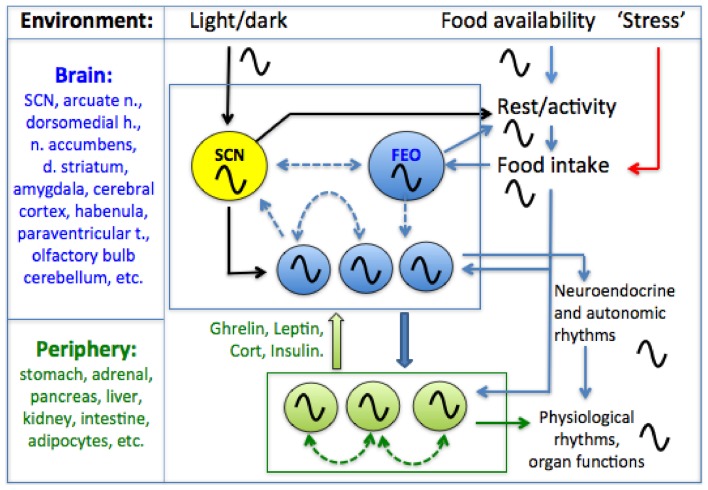
**A conceptual model of the mammalian circadian system**. A circadian pacemaker located in the retinorecipient suprachiasmatic nucleus (SCN) in the hypothalamus generates circadian rest-activity, feeding, body temperature and other rhythms that entrain to environmental light-dark (LD) cycles. The daily feeding rhythm provides time cues that entrain circadian oscillators elsewhere in the brain and in most peripheral organs and tissues. Limiting food access to a single daily meal entrains central and peripheral oscillators, decoupling these from the SCN pacemaker which remains entrained to LD. Some SCN-independent food-entrainable oscillators (FEOs) drive daily rhythms of food anticipatory activity. The location of these FEOs and the signals that entrain them to mealtime, remain to be determined. Peripheral clocks could entrain central FEOs via hormonal pathways involving ghrelin, leptin, corticosterone and insulin, although none of these alone are required for food-anticipatory activity rhythms. Solid arrows indicated interactions for which empirical evidence exists, while dashed arrows indicate possible interactions (coupling among oscillators in different structures). For simplicity, some other possible interactions among and between central and peripheral clocks and meal-related cues are omitted.

Behavior and physiology are regulated by a hierarchically organized system of circadian oscillators located in the brain and in most peripheral tissues and organs (Figure 1). Circadian timekeeping coordinates cellular and physiological processes internally, and synchronizes these with daily cycles in the environment, ensuring that biological activities, from gene expression to foraging behavior, occur in the right sequence and at the right time of day. Although daily light-dark (LD) cycles are considered to be the dominant environmental stimulus for synchronizing circadian oscillators to local time, for many circadian processes, it is the timing of food intake that is most important. In this review, we briefly outline the evidence that feeding patterns regulate circadian clocks in mammals. We then critically examine the role of metabolic and stress-related hormones in this process. An important objective for the purposes of this Frontiers Topic (neuroendocrine mechanisms that connect feeding behavior and stress) is to draw attention to chronobiological concepts that provide a framework for understanding how anything that can affect feeding behavior, including acute or chronic stress stimuli (Maniam and Morris, 2012; Patterson and Abizaid, 2013; Sinha and Jastreboff, 2013), can potentially alter circadian organization within the brain and in peripheral organs and tissues. Whether the consequences for the organism are adaptive or pathological may depend on the temporal parameters of feeding behavior.

## Meal timing entrains circadian clocks

The role of food intake in the regulation of circadian clocks was first revealed by studies of circadian activity rhythms in food-restricted rodents. Acute and chronic food restriction elicit well-known behavioral and physiological responses to maintain metabolic homeostasis. Acute deprivation recruits neural circuits that promote arousal and food seeking behavior. Chronic caloric restriction induces physiological adaptations to facilitate the extraction and storage of energy from ingested nutrients and to reduce energy expenditure. If caloric restriction is chronic and food availability is limited to a particular time of day, further adaptations can occur in the temporal regulation of metabolism and food seeking behavior. These adaptations involve entrainment of circadian clocks in the brain and in peripheral organs and tissues by stimuli associated with food intake.

Physiology and behavior are regulated by circadian clocks that induce daily rhythms in synchrony with environmental cycles. In mammals, a master circadian clock is located in the suprachiasmatic nucleus (SCN) of the anterior hypothalamus (Weaver, 1998; Welsh et al., 2010). The SCN contains a large population of circadian oscillator cells that are entrained to daily LD cycles via a direct input from intrinsically photoreceptive retinal ganglion cells. SCN ablation eliminates circadian organization of behavior and physiology if food is freely available. Circadian oscillators also exist in other brain regions and most if not all peripheral organs and tissues (Yamazaki et al., 2000; Guilding and Piggins, 2007; Dibner et al., 2010). Within each tissue, circadian oscillator cells must be coupled with each other for circadian organization to emerge at the tissue level. The SCN plays a special role within this multioscillatory system by providing signals that maintain coupling of oscillators within most tissues. SCN timing signals are conveyed by neural (autonomic), hormonal (hypothalamo-pituitary) and behavioral pathways (Dibner et al., 2010). Of particular importance is SCN control of the daily rhythm of feeding behavior. A dominant role for food intake in the control of peripheral rhythms is readily demonstrated by restricting rats and mice to a 2–6 h mealtime in their usual rest phase (the lights-on period), when they normally eat very little. Scheduled daytime feeding shifts the timing of clock gene rhythms and functional rhythms in most peripheral organs and tissues to realign with expected mealtime (Boulos and Terman, 1980; Dibner et al., 2010). If a daily LD cycle is present, the SCN itself is not shifted by daytime feeding schedules (Damiola et al., 2000; Hara et al., 2001a; Stokkan et al., 2001). Dissociation of peripheral oscillators from the SCN during restricted daytime feeding suggests that the SCN normally coordinates daily rhythms of physiology by its role as the pacemaker for feeding behavior.

**Figure 2:**
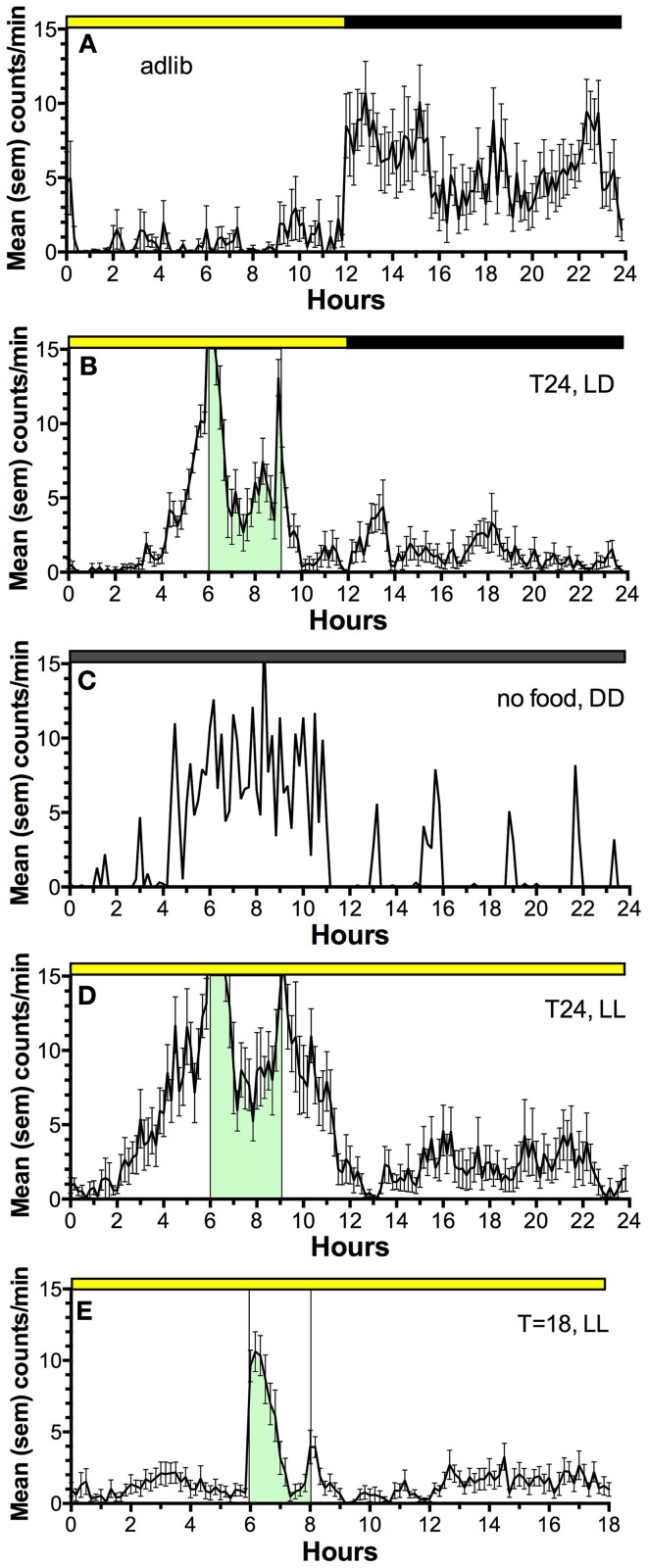
**Average waves illustrating the temporal distribution of spontaneous locomotor activity of a representative rat under the following feeding conditions. (A)** *ad-lib* access to food (10 day average). **(B)** Food restricted to 3-h/day, beginning 6 h after lights on, for 3 weeks (last 10 day average). **(C)** Day 2 of total food deprivation after 3 weeks of restricted daytime feeding. **(D)** Food restricted to 3-h/day for 3 weeks, after a month in constant light with food *ad-lib*, to eliminate the light-entrainable, free-running rhythm controlled by the SCN (a reversible SCN “lesion” procedure). **(E)** Food restricted to 2-h/day in constant light, delivered every 18 h, illustrating failure of rats to anticipate a daily meal at regular but non-circadian intervals. Mealtime is denoted by green shading and vertical bars. Activity was measured by motion sensors, and plotted as average counts per minute, in 10 min bins.

Restricted daytime feeding also induces a behavioral rhythm of food seeking behavior that anticipates the daily mealtime (Figure 2) (Boulos and Terman, 1980; Mistlberger, 1994; Stephan, 2002). If the SCN are ablated and food is available *ad-libitum*, behavior and physiology lose circadian organization. However, if food is provided once every 24 h in a reduced amount, food anticipatory activity rhythms emerge and circadian rhythms of physiology are restored (Stephan et al., 1979; Boulos et al., 1980). The timing of food intake is thus fundamental to circadian organization of behavior and physiology and involves effects of food on circadian oscillators downstream from the master, LD-entrained circadian pacemaker in the SCN.

Despite the significance of food as a “zeitgeber” (entrainment cue) for circadian regulation of food-anticipatory behavior, the neural and molecular mechanisms of this SCN-independent circadian function are not yet well-understood. The daily rhythm of food anticipatory activity exhibits canonical properties of circadian clock control (Boulos and Terman, 1980; Mistlberger, 1994; Stephan, 2002). Once established, the rhythm persists for several cycles during total food deprivation (Figure 2C); the timing mechanism is therefore self-sustaining, unlike simple hourglass or interval timers (e.g., like a stomach that gradually empties after a large meal). If the feeding schedule differs greatly from 24 h (e.g., is <22 or >30 h) the food anticipation rhythm does not emerge (Figure 2E); it has so-called “limits to entrainment” that suggest involvement of an oscillator with an intrinsic periodicity in the circadian (~24 h) range. If the mealtime is shifted by 6 h or more to a new time of day, the food anticipation rhythm takes several days to realign with mealtime. These properties have been interpreted as evidence that one or more correlates of food intake entrain a circadian oscillator (or population of oscillators) that determines the daily timing of foraging activity. Given that circadian food anticipatory rhythms persist in SCN-ablated rats and mice, food-entrainable oscillators (FEOs) hypothesized to drive anticipatory behavior must be located elsewhere in the brain or body. There are many candidate sites, because, as noted, daily feeding schedules synchronize circadian oscillators in many brain regions and virtually every organ system (Feillet et al., 2006, 2008; Verwey and Amir, 2009; Dibner et al., 2010). Lesion studies have so far failed to confirm a single critical locus in the brain (Mistlberger, 1994, 2011; Davidson, 2009), suggesting that circadian timing of food anticipatory activity involves an anatomically distributed population of FEOs. It is also likely that there are multiple stimuli associated with scheduled feeding that are capable of functioning as zeitgebers for entraining FEOs.

In the following sections, we evaluate a potential role for metabolic hormones as signals by which FEOs are coupled to daily feeding schedules, or by which FEOs broadcast timing information to neural circuits that organize food seeking behavior. Although the primary emphasis is on food entrained circadian regulation of behavior, these hormonal signals may also play a role synchronizing circadian clocks and functions in peripheral organs (Dibner et al., 2010). The light-entrainable circadian pacemaker in the SCN, as noted, remains entrained to LD cycles regardless of the timing of food intake. There are limited circumstances under which its phase can be controlled or modified by mealtime (e.g., in constant dark or light; Abe et al., 1989; Mistlberger, 1993; Challet, 2010), but neural and hormonal signals that mediate these effects are little studied. Consequently, the literature reviewed here concerns circadian oscillators located outside of the SCN pacemaker.

To be considered a candidate as an entrainment signal for food anticipatory circadian rhythms, a hormone (or any stimulus factor) should exhibit a daily rhythm that is induced by or is synchronous with scheduled feeding. The factor should be capable of shifting the phase of one or more food-entrained rhythms (a requirement for co-activation by 2 or more factors is possible). Blocking the factor may prevent shifting of one or more food entrained rhythms (unless there are multiple redundant signals). To be considered a candidate as an output of FEOs responsible for driving food anticipatory activity (or other food entrained rhythms) the factor should exhibit a daily rhythm that correlates strongly with locomotor activity (or other rhythms), under a variety of conditions (e.g., during total food deprivation, when food anticipatory rhythms established by scheduled feeding persist for several cycles despite the absence of a daily meal). Enhanced expression of the factor may increase the amplitude of food anticipatory activity (or other rhythms), while blocking the factor should attenuate or prevent expression of food anticipatory activity (or other rhythms). Attenuation could be modest or negligible if there are multiple redundant output signals. Food anticipation could also be enhanced or attenuated by factors that are downstream from FEOs, or that converge downstream via other pathways. Therefore, the observation that manipulation of a factor alters the amount of food anticipatory activity does not alone permit a strong conclusion that the factor is either an input or output of FEOs.

## Peripheral hormonal mechanisms

### Corticosterone

The synthesis and secretion of corticosterone (CORT) from the adrenal gland is under control of descending neural (autonomic) and hormonal (adrenocorticotropin) signals, interacting with circadian oscillators intrinsic to cells of the adrenal gland (Kaneko et al., 1981; Oster et al., 2006). Daily restricted feeding markedly alters the circadian profile of circulating CORT levels (Krieger, 1974). In nocturnal rodents under ad lib feeding conditions CORT levels peak close to dark onset. When food is restricted to the middle of the light phase, the daily rhythm of CORT secretion becomes bimodal, with one peak occurring at the expected mealtime and a second at the beginning of the dark period (Krieger, 1974). Treatment with sodium pentobarbital can suppress the pre-feeding peak while sparing the nocturnal peak, suggesting that these two peaks are stimulated by separate neural mechanisms (Honma et al., 1984a).

The correlated rise of CORT release and food seeking activity prior to mealtime raises the possibility that CORT may function as an endogenous entrainment signal for FEOs or as an output from FEOs to behavior. However, preprandial CORT secretion has been ruled out as a signal involved in food anticipatory behavioral rhythms, as adrenalectomized rats display apparently normal food anticipatory activity (Stephan et al., 1979; Boulos et al., 1980; Segall et al., 2008; Sujino et al., 2012). The available evidence reveals no obvious difference in the rate at which anticipation emerges or in the magnitude or duration of steady state anticipatory activity in adrenalectomized rats. This conclusion is supported by studies of food anticipation in rats provided with two daily meals separated by 6 h or more. Under these schedules rat readily anticipate both meals and continue to concentrate activity at both expected mealtimes during total food deprivation tests. The properties of each bout of anticipation suggest involvement of two independently entrained FEOs (Stephan, 1989; Mistlberger et al., 2012). At a 6 h inter-meal interval, CORT secretion rises in anticipation of the first meal, but does not rise again until near the end of the second meal (Honma et al., 1984b). When the meals were omitted only the peak to the first meal was observed, thereby dissociating CORT secretion from behavioral anticipation of the second meal.

Circadian oscillators in peripheral organs, including the adrenal gland, liver, stomach, intestines, pancreas, kidney, heart, and lungs, are entrained by daily feeding schedules (Damiola et al., 2000; Stokkan et al., 2001; Davidson et al., 2003). Circadian clock gene oscillations in many of these tissues can also be shifted by glucocorticoid administration (Balsalobre et al., 2000; Reddy et al., 2007; Pezuk et al., 2012) or by adrenalectomy (Pezuk et al., 2012), suggesting a role for glucocorticoids in phase control of peripheral clocks during adlib food access, and potentially also during restricted feeding. A strong form of the latter hypothesis, that glucocorticoids may be necessary for resetting of peripheral tissues during restricted feeding, is refuted by observations that peripheral clocks shift more rapidly in response to daytime feeding in adrenalectomized mice or knockout mice lacking hepatic glucocorticoid receptors (Balsalobre et al., 2000; LeMinh et al., 2001). This indicates that nocturnal glucocorticoid secretion normally reinforces circadian organization of peripheral clocks in rodents with adlib food access, by opposing rapid phase shifts that might be induced by transient alterations of food access. This role may be more prominent for some tissues. Circadian clock gene rhythms in the liver are not shifted by a daily CORT injection meant to simulate a mid-day feeding schedule (Stokkan et al., 2001) and shifting of the liver clock by mid-day feeding is not impeded by a late-day CORT injection meant to simulate the normal nocturnal rise of CORT. By contrast, shifting of circadian clocks in the kidney and lungs to restricted feeding is blocked by a late day CORT injection. These findings indicate that peripheral oscillators likely respond to multiple feeding-related signals in a tissue specific manner.

### Ghrelin

Acyl-ghrelin and des-acyl ghrelin are peptide hormones derived from preproghrelin, which is synthesized by oxyntic cells of the stomach (Kojima et al., 1999) and by neurons in medial and lateral hypothalamic nuclei (Cowley et al., 2003). Systemic and intracerebroventricular (ICV) administration of acyl-ghrelin stimulates feeding in rats and mice (Nakazato et al., 2001; Toshinai et al., 2006). Des-acyl ghrelin may also stimulate feeding if administered (ICV), but not systemically (Toshinai et al., 2006). The orexigenic action of gastric ghrelin is mediated primarily by activation of agouti-related peptide and neuropeptide Y (AgRP/NPY) neurons in the arcuate nucleus (ARC), resulting in release of AgRP and NPY. Ghrelin also exerts an inhibitory action at proopiomelanocortin (POMC) neurons, preventing release of anoxerigenic peptides. The growth hormone secretagogue 1 receptor (GHSR) is the only known ghrelin receptor and is found in high levels in the ARC. GHSR mRNA has also been localized to multiple hypothalamic areas involved in feeding and arousal and extra-hypothalamic regions including the hippocampus, ventral tegmental area (VTA), substantia nigra (SN), tuberomammilary nucleus (TMN), Edinger-Westphal nucleus (EN), dorsal and median raphe nuclei, laterodorsal tegmental nuclei and the facial nucleus (Guan et al., 1997).

Plasma ghrelin increases prior to scheduled meals in food restricted rats and mice (Bodosi et al., 2004; Drazen et al., 2006; LeSauter et al., 2009) and prior to scheduled or habitual mealtimes in humans (Cummings et al., 2001; Frecka and Mattes, 2008). Gastric oxyntic cells exhibit a daily rhythm of circadian clock gene expression that is entrained by restricted feeding, suggesting that food-anticipatory release of gastric ghrelin is a function of gastric FEOs (LeSauter et al., 2009). The food-entrainable rhythmicity and orexigenic properties of gastric ghrelin present an intriguing circumstantial case that gastric ghrelin may participate in the regulation of circadian food anticipatory rhythms, either by stimulating appetite and activity or by entraining central clocks that drive behavioral rhythms. This hypothesis can be evaluated by the results of correlation, stimulation and gene knockout studies.

Plasma ghrelin and locomotor activity exhibit a correlated rise prior to a scheduled daily meal but become dissociated during subsequent total food deprivation tests. Food entrained activity recurs at the expected mealtime for at least 4 days without food (Boulos et al., 1980; Mistlberger, 1994). This property is critical to the interpretation that anticipation of a daily meal is based on entrainment of a self-sustaining circadian clock. Acyl-ghrelin also rises prior to the first skipped meal, but beyond a day of fasting decreases and remains at low levels. Des-acyl ghrelin, which does not stimulate feeding or activity following systemic administration, is responsible for the rise of total plasma ghrelin evident during extended fasting (Liu et al., 2008; Kirchner et al., 2009). A similar dissociation between acyl-ghrelin and food anticipatory activity during food deprivation tests is evident in rats fed two daily meals separated by 12 h (Patton et al., 2012). During the first 24 h without food, ghrelin levels rise in anticipation of the first mealtime but then remain elevated for the rest of the day, unlike locomotor activity, which decreases after the first meal before rising prior to the second mealtime 10 h later (Patton et al., 2012). A third dissociation can be inferred from studies of clock gene rhythms in the stomach of rats entrained to a midday meal for several weeks, and then allowed ad-lib food access for a week. If the food is then removed for 2–4 days, the rats become active at the previous mid-day mealtime. The gastric circadian clock, by contrast, is shifted by daytime feeding, but reverts to its original phase after a few days of adlib feeding (Davidson et al., 2003). If gastric ghrelin secretion is controlled by gastric circadian oscillators, then plasma ghrelin should rise at night and not in the middle of the day when food anticipatory activity reappears during deprivation tests.

Although systemic administration of acyl-ghrelin stimulates food intake, its effect on locomotor activity is less clear. In mice, central administration of acyl-ghrelin increased eating, activity and wake time, while peripheral ghrelin stimulated food intake but did not alter either wake time or locomotor activity for 1 and 6 h post-injection (Szentirmai, 2012). At orexigenic doses, systemically administered ghrelin was not sufficient to stimulate activity, as predicted if gastric ghrelin were responsible for circadian food anticipatory activity.

**Figure 3:**
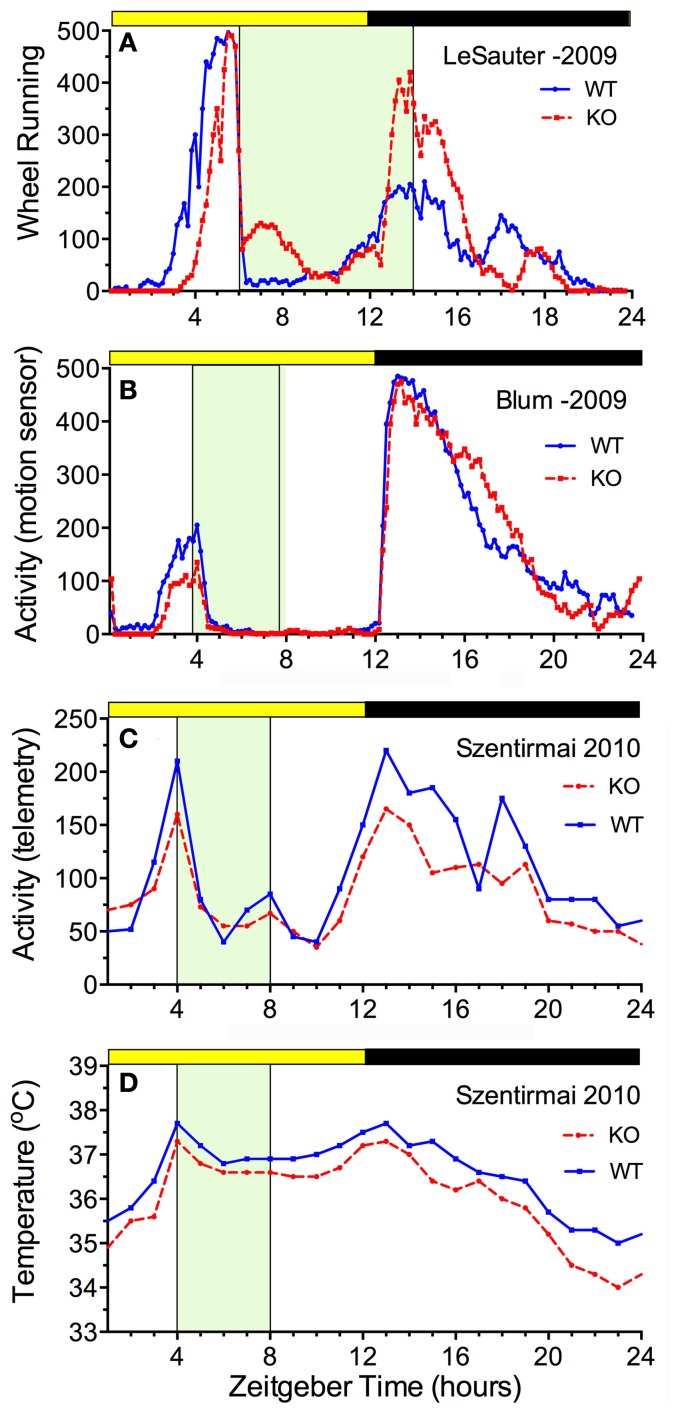
**Average waves illustrating the consequences of ghrelin receptor knockout (A,B) and preproghrelin KO (C,D) on food-entrained locomotor activity and body temperature in mice**. Knockout groups are represented by the red dashed lines, WT by the solid blue lines. **(A)** The onset of wheel running activity is modestly delayed in ghrelin receptor knockout mice, but rises to the same level (LeSauter et al., 2009). **(B)** The onset of running is not delayed but rises to a lower level in ghrelin receptor knockout mice (Blum et al., 2009). **(C)** Locomotor activity measured by telemetry is slightly reduced at all time points in preproghrelin KO mice compared to WT mice, but increases equivalently prior to a 4-h daily meal in both groups (Szentirmai et al., 2010). **(D)** The same results were obtained for core body temperature measured by telemetry (Szentirmai et al., 2010).

The availability of ghrelin ligand and receptor knock-out mice permits a direct assessment of whether ghrelin is necessary for food anticipatory activity. The results of 5 studies are consistent in demonstrating that mice do not require ghrelin signaling to anticipate a daily meal (Figure 3). Three of these studies reported food anticipatory activity and body temperature in ghrelin ligand knockout mice (Szentirmai et al., 2010; Gunapala et al., 2011) (Figures 3C,D) or ghrelin receptor KO mice (Gooley et al., 2006) that did not differ from anticipatory activity in WT mice. Two other studies also reported significant FAA in ghrelin receptor KO mice, but with alterations relative to WT mice. In one case, the average duration of FAA was decreased (i.e., the onset was closer to mealtime) without a change in the peak level (LeSauter et al., 2009) (Figure 3A), while in the other case, the peak level was reduced with little change in FAA duration (Blum et al., 2009) (Figure 3B).

In contrast to these 5 studies, there are two reports that ghrelin receptor knock out mice do not anticipate a daily meal. In one of these studies, food anticipation was quantified as the total number of beam breaks 2-h prior to a 4-h daily mealtime, during one baseline day and on day 14 of food restriction in a separate recording cage (Davis et al., 2011). Raw data (24 h activity waveforms) were not provided, and without normalizing for total daily activity, comparing counts in a 2-h time bin does not provide information about the presence or absence of anticipation. Also, the mealtime, relative to the LD cycle was not specified. Consequently, the “evidence for absence” in this study is weak. The other study evaluated the effects of ghrelin receptor knockout on hyperactivity in mice induced by severe caloric restriction, in an “activity based anorexia” protocol (a 2-h daily meal provided at lights-off for 3-days; Verhagen et al., 2011a). WT mice exhibited daytime hyperactivity, which was absent in the KO mice. However, activity was maximal in the middle of the light period in both groups and decreased as the nocturnal mealtime approached, indicating that hyperactivity during the first 3 days of extreme caloric restriction in the WT mice was non-specific rather than food-anticipatory. When food is acutely restricted to a few hours/day, mice may exhibit hyperactivity for the first few days, which dissipates as the amount of food consumed during the mealtime increases, and a stable pattern of food anticipatory activity, peaking at expected mealtime time, emerges. If the caloric restriction is too severe, the hyperactivity does not dissipate, appetite is suppressed and metabolic collapse may ensue.

Collectively, the results of ghrelin knockout studies indicate that ghrelin secretion is not required for anticipation of a daily meal. If ghrelin does participate in circadian expression of food anticipatory activity under some experimental conditions, it is likely acting downstream from or in parallel with FEOs hypothesized to drive FAA, or it is redundant with other FEOs or with other signals that can entrain FEOs. The site of action of ghrelin may be both hypothalamic and mesocorticolimbic, given recent observations that ghrelin receptor KO mice exhibit lower levels of food-anticipatory cFos expression (a correlate of neuronal activity) in lateral hypothalamic orexin neurons, the VTA and the nucleus accumbens shell (Lamont et al., 2012). Whether ghrelin is responsible for controlling the phase of circadian oscillators in these areas, or in any peripheral tissues, has not yet been demonstrated.

### Ghrelin and anticipation of palatable food

Food anticipation can also be observed in rats and mice without caloric restriction, by providing access to a palatable snack (e.g., chocolate) at a fixed time of day, with regular chow available ad-libitum (Mistlberger and Rusak, 1987; Mendoza et al., 2005; Hsu et al., 2010). Palatable feeding schedules are associated with induction of *cFos* prior to snack time in cortical and limbic structures involved in reward and motivation (n. accumbens core and shell, central nucleus of the amygdala, prefontal cortex). All of these areas express circadian rhythms of clock gene expression, and are innervated by dopaminergic projections from the VTA (Webb et al., 2009a). Neural activity in the VTA exhibits a daily rhythm, but clock gene expression in this area is constitutive rather than circadian, indicating that VTA rhythmicity is driven by inputs (Webb et al., 2009b; Moorman and Aston-Jones, 2010; Baltazar et al., 2013). A candidate rhythmic input is ghrelin, which increases the firing rate of VTA neurons that release DA in the VTA (Abizaid et al., 2006). This raises the possibility that ghrelin, by activating DA neurons in the VTA that innervate circadian oscillators in the limbic forebrain, might play a role as an entraining stimulus for daily rhythms of palatable food anticipation. If so, then ghrelin levels should rise in anticipation of a palatable meal, and ghrelin knockout or receptor antagonists should eliminate or attenuate palatable food anticipation.

Studies of plasma ghrelin levels in rats anticipating a daily palatable meal have yielded mixed results. One study reported that plasma ghrelin, sampled at the expected time of chocolate delivery (6 h after lights-on in LD 12:12), correlated positively with locomotor activity counts during the preceding 3 h (Merkestein et al., 2012). Ghrelin levels in these rats were intermediate between ghrelin levels in adlib fed rats and in rats restricted to a single daily meal at this time of day. A second study reported that plasma ghrelin was significantly increased 30 and 60 min prior to a daily 6 h meal consisting of regular chow or high fat chow, but was not elevated at 30, 60, and 90 min time points prior to 2-h daily access to chocolate (5 h after lights-on), with regular chow available *ad-libitum* (Dailey et al., 2012). Rats in all 3 groups exhibited significant anticipatory activity, although the amount of anticipatory activity in rats receiving chocolate without food restriction was about half of that in the food restricted groups. These results indicate that elevated ghrelin is not necessary for the induction of anticipatory activity, but may promote its expression. An explanation for the different results in the two studies remains to be established, but could be related to the parameters of the feeding schedules (meal time and duration) or small differences in the blood sampling time points.

Anticipation of palatable foods without caloric restriction has not yet been assessed in ghrelin deficient mice. However, the ghrelin receptor antagonist JMV2959, administered i.c.v. 3-h prior to chocolate access, dose-dependently attenuated anticipatory activity by 40–60%, while administration of acyl-ghrelin increased anticipatory activity by about 50% (Merkestein et al., 2012). These findings are consistent with a role for ghrelin as a modulatory factor in palatable meal anticipation.

### Leptin

Leptin is an anorexigenic hormone released from adipose tissue in proportion to lipid stores (Zhang et al., 1994). Leptin acts in opposition to ghrelin at the ARC, inhibiting NPY/AgRP neurons and stimulating POMC/CART neurons, resulting in decreased food intake and increased metabolic rate (Kageyama et al., 2012). Serum leptin shows a daily rhythm in humans and mice, increasing during the waking/feeding period and decreasing during the sleeping/fasting period (Sinha et al., 1996; Ahima et al., 1998; Ahren, 2000; Shea et al., 2005). In humans, the rhythm is the net result of daily variations in food intake (leptin increases after feeding and decreases during fasting) and an endogenous clock (Shea et al., 2005). In mice, leptin synthesis and secretion are also subject to circadian regulation, at least in part by circadian oscillators intrinsic to adipocytes (Zvonic et al., 2006; Otway et al., 2009). However, expression of the daily rhythm of circulating leptin appears to be dependent on food intake. The rhythms of plasma leptin, and the adipocyte clock (Zvonic et al., 2006), are inverted if food is restricted to the middle of the light period, but the plasma leptin rhythm is abolished during total food deprivation (Ahima et al., 1998; Elimam and Marcus, 2002; Martinez-Merlos et al., 2004). Food anticipatory activity rhythms, as noted, persist robustly during several days of total food deprivation. This dissociation between circulating leptin levels and food anticipatory rhythms during food deprivation indicates that variations in plasma leptin do not drive the daily rhythm of food anticipatory behavior.

Consistent with this interpretation, leptin insensitive Zucker rats and leptin deficient ob:ob mice are obese and hypoactive when food is available ad-lib, but exhibit strong FAA when restricted to a single mid-day meal and the amount of FAA is increased relative to WT controls (Mistlberger and Marchant, 1999b; Ribeiro et al., 2011). Conversely, systemic administration of leptin reduces pre-meal activity in food-restricted rats/mice (Verhagen et al., 2011b). These results indicate that leptin, like ghrelin, plays a modulatory role in the expression of food anticipatory activity, consistent with its role in modulating food intake under free-feeding conditions.

### Insulin and glucagon

Homeostasis of plasma glucose is critically dependent on the pancreatic hormones insulin and glucagon. Food ingestion is the major stimulus for insulin secretion, but external stimuli that predict food intake also play an important role stimulating release (Woods et al., 1970). In the scheduled feeding paradigm, time of day (circadian phase) has been conceptualized as a conditioned stimulus for anticipatory insulin secretion (Woods et al., 1977). It is now established that the pancreas contains autonomous circadian oscillators that are reset by restricted daytime feeding, independently of the master clock in the SCN (Damiola et al., 2000). Insulin secreting beta cells are among those thought to contain an intrinsic circadian clock (Marcheva et al., 2010; Sadacca et al., 2011). Therefore, any increase in plasma insulin that occurs in rats at an expected daily mealtime (Wiley and Leveille, 1970; Woods et al., 1977; Drazen et al., 2006; Vahl et al., 2010) presumably reflects entrainment of local pancreatic oscillators, a process distinct from the central neural mechanisms that mediate stimulus-response conditioning of insulin release.

A food anticipatory increase in plasma insulin has not always been reported (e.g., Davidson and Stephan, 1999; Diaz-Munoz et al., 2000; Frecka and Mattes, 2008), This may be due to sampling time and resolution, as significant increases when reported appear to occur very close to mealtime (e.g., within 15 min; Drazen et al., 2006; Vahl et al., 2010). This raises the possibility that preprandial insulin release in some studies is a conditioned response to noises made by research staff arriving in advance to deliver the daily meal (we have observed lever pressing for food conditioned to the sound of a door opening to enter an anteroom outside of an animal recording room, a few minutes prior to a daily mealtime; Peterson and Mistlberger, unpublished observations). Whatever the explanation for the different results across studies, the proximity of preprandial insulin release to mealtime rules out insulin as an initiator of food anticipatory activity, which typically begins 2–3 h before mealtime. It is also appears that prandial insulin release is not required for entrainment of oscillators driving FAA, because streptozotocine-induced diabetes in mice does not prevent induction of food anticipatory activity or resetting of circadian clocks in the liver, heart or kidney by restricted daytime feeding (Davidson et al., 2002; Oishi et al., 2004).

Glucagon, the other major pancreatic hormone, normally varies inversely with insulin. Two studies reported that plasma glucagon levels decrease in the hours preceding a scheduled daytime meal in rats (Davidson and Stephan, 1999; Diaz-Munoz et al., 2000). It has not been established that pancreatic alpha cells that produce glucagon possess an intrinsic circadian clock, and there is no evidence that glucagon participates in food anticipatory behavioral rhythms.

### Glucagon like peptide 1

Glucagon like peptide 1 (GLP-1) is synthesized in the periphery and in the brain. In the periphery GLP-1 is derived from preproglucagon synthesized in L cells of the intestine and alpha cells of the pancreas (Mojsov et al., 1990). In the CNS GLP-1 is produced in the nucleus of the solitary tract, the dorsal and central reticular nucleus, the PVN, DMH and to a lesser extent the ARC (Larsen et al., 1997). GLP-1 receptors are widely expressed in the CNS with large numbers in the ARC, PVN, and SON, and fewer in the lateral preoptic area, PVN, LH, bed nucleus of the stria terminalis and DMH (Shughrue et al., 1996). Most regions that express GLP-1 receptors are behind the BBB, and whether peripheral GLP-1 has access to these receptors is under debate (Trapp and Hisadome, 2011). It is also not clear if post-prandial peripherally released GLP-1 signals for the release of central GLP-1.

Two studies have investigated the effects of restricted feeding schedules on GLP-1 secretion. In one study, rats were maintained on a 4-h daily meal beginning 5-h after lights-on for 14 days. Plasma GLP-1 was found to increase 4-fold over an interval from 75 to 60 min before the meal, and then decrease back to baseline levels 30 min before mealtime (Vahl et al., 2010). In a second study, rats anticipating a 6-h daily meal beginning 3-h after lights-on also exhibited increased GLP-1 at 90 and 60 min before mealtime, and no difference at 30 min prior to the meal, by comparison with adlib fed rats. By contrast, a group of rats anticipating 2-h access to chocolate, without caloric restriction, showed no change in GLP-1 at 90, 60, or 30 min time points before feeding (Dailey et al., 2012). These results indicate that a premeal peak of GLP-1 is not necessary for anticipation of a daily palatable meal in the absence of caloric restriction. Whether the onset of GLP-1 secretion prior to a single daily meal in calorically restricted rats correlates with the onset of anticipatory activity has not been assessed. Sustained increments of GLP-1 are clearly not required to produce the continuous food anticipatory activity typical of food-entrained rodents, but it is conceivable that an early GLP-1 spike is part of a cascade of responses that gate the onset of food anticipatory activity or entrain FEOs driving food anticipation.

## Central neuropeptides

### Melanocortins and neuropeptide Y

Circulating ghrelin and leptin exert opposite effects on the expression of circadian food anticipatory activity, and may do this at least in part by differential effects on the central melanocortin system. The melanocortin system consists of separate populations of neurons in the arcuate nucleus that release ligands for melanocortin-3 (McR3) and melanocortin-4 (McR4) receptors. One population of arcuate neurons synthesizes POMC, a precursor for α, β, and γ-melanocortin-stimulating hormones (MSH) which function as agonists at Mc3R and Mc4R receptors (Mountjoy, 2010). A second population of arcuate neurons corelease AgRP and NPY. AgRP acts as an inverse agonist at melanocortin receptors (Cone, 2005). Mc3R receptors are predominantly expressed in hypothalamic and limbic structures, with high levels in areas implicated in feeding and reward (VMH and VTA) (Roselli-Rehfuss et al., 1993), while Mc4R are more widely distributed within the brain.

The melanocortin system plays a critical role in the regulation of metabolism and feeding. α-MSH potently inhibits feeding (Fan et al., 1997), whereas AgRP stimulates feeding (Small et al., 2001; Aponte et al., 2011; Krashes et al., 2011). Deletion of POMC and Mc3R and Mc4R receptors induces hyperphagia, obesity, hyperinsulinemia, insulin insensitivity and loss of response to leptin (Barsh and Schwartz, 2002; Sutton et al., 2006), whereas AgRP deletions induce anorexia and weight loss and eliminate the orexigenic response to ghrelin (Chen et al., 2005; Gropp et al., 2005; Luguet et al., 2005; Aponte et al., 2011). Given the effects of ghrelin and leptin deficiency on food anticipatory activity rhythms, a prediction is that melanocortin receptor deficient mice should also exhibit a food anticipation phenotype. Mc4^−/−^ mice exhibit normal FAA, but Mc3^−/−^ mice have been reported to exhibit significantly attenuated food anticipatory rhythms of wheel running activity and waking (Butler et al., 2000; Sutton et al., 2008). However, the data fall short of supporting a strong conclusion that Mc3 receptors are “required” for entrainment of behavioral rhythms to feeding schedules, given that anticipation was evident in KO mice when recordings were made for longer than a few days (Sutton et al., 2008). Also, normal levels of food anticipatory activity have been observed in Mc3R^−/−^ mice assessed by video based analyses (Steele, pers. commun.) and enhanced levels have been reported in mice lacking both Mc3 receptors and leptin assessed (Ribeiro et al., 2011). Whether differences in the amount of anticipatory activity observed in Mc3R^−/−^ mice studied in different labs is related to methodological differences (e.g., recording devices) or to the use of knockout mice obtained from different sources remains to be evaluated.

AgRP neurons co-release the potent orexigen NPY. Synthesis and release of NPY in the rat hypothalamus exhibits a daily rhythm with a peak prior to the nocturnal feeding period in adlib fed rats and prior to a schedule daily meal in food restricted rats (Akabashi et al., 1994; Xu et al., 1999; Yoshihara et al., 1996). Central administration of NPY induces behavioral and endocrine responses characteristic of rats anticipating a daily meal, including secretion of insulin and corticosterone and increased locomotor activity (Stanley and Leibowitz, 1984; Smialowska et al., 1994; Sainsbury et al., 1997). Despite this functional profile, NPY appears to be dispensable for induction of food anticipatory activity rhythms. NPY KO mice show reduced food anticipatory activity on day 7 of restricted daytime feeding but normal food anticipation by day 14 (Gunapala et al., 2011). Treatment of neonatal rats with monosodium glutamate severely reduces NPY immunoreactivity in the arcuate nucleus, but does not attenuate food anticipatory activity in rats (Mistlberger and Antle, 1999a). Radiofrequency lesions of the hypothalamic paraventricular nucleus, a hot spot for orexigenic effects of NPY, also do not impair food anticipatory activity in rats (Mistlberger and Rusak, 1988).

### Orexin/hypocretin

Orexin A and B (hypocretin 1 and 2) are derived from prepro-orexin synthesized exclusively in a population of neurons distributed within the dorsomedial, perifornical and lateral hypothalamic areas (Date et al., 1999). Orexin neurons project widely throughout the hypothalamus, thalamus, brain stem, and forebrain (Peyron et al., 1998). Orexins act via two G-protein coupled receptors. OX1R is selective for orexin A whereas OX2R binds both orexin A and B with similar affinity. When administered ICV to rats and mice, orexin A acutely stimulates feeding (Lubkin and Stricker-Krongrad, 1998; Sakurai et al., 1998), plasma corticosterone, wakefulness (Hagan et al., 1999), and locomotion (Nakamura et al., 2000). Orexin neurons are activated by a variety of stimuli, including hypoglycemia, caloric restriction, ghrelin administration and non-specific arousal (Sakurai et al., 1998; Cai et al., 1999; Yamanaka et al., 2003; Webb et al., 2008). Conversely, antagonism of ORX1R reduces food intake and body weight (Smart et al., 2002). Restricted feeding has been found to increase FOS staining in orexin neurons in the LHA in a manner similar to glucose-deprivation (Kurose et al., 2002) and in anticipation of a daily meal (Akiyama et al., 2004).

**Figure 4:**
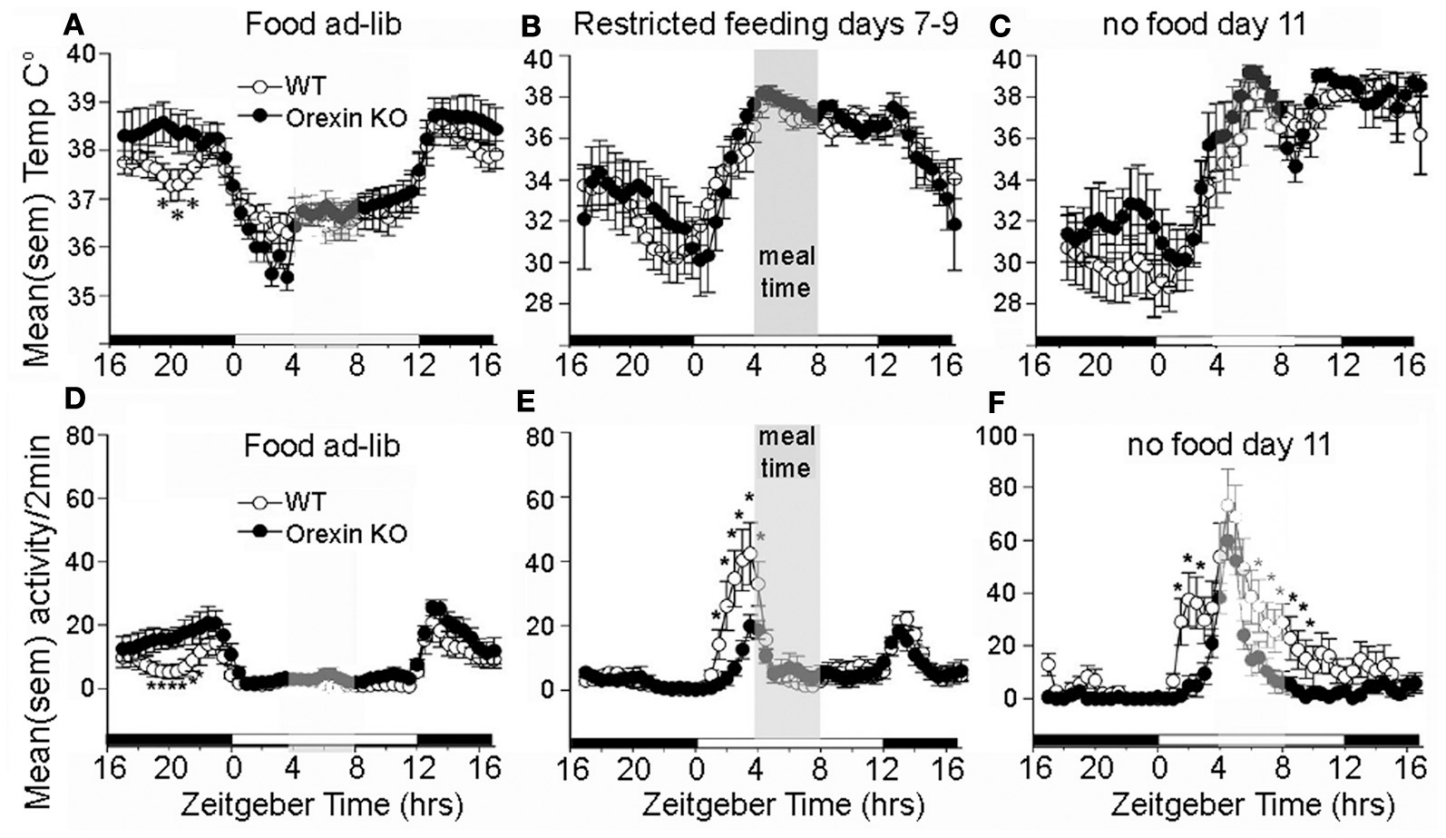
**Average waves illustrating daily rhythms of core body temperature (A–C) and locomotor activity (D–F) in WT mice (open circles) and orexin KO mice (closed circles)**. WT and KO mice show an equivalent anticipatory rise of body temperature prior to a 4-h daily meal **(B)** and this rhythm persists for at least 2 days of total food deprivation. **(C)** By contrast with WT mice, the KO mice exhibit attenuated anticipatory activity during days 7–9 of restricted feeding, but show an equivalent rise of activity at the expected mealtime during the second day without food, indicating that the timing mechanism for food anticipation is present despite the absence of orexin signaling. ^*^significant *p* < 0.01 between WT and KO. Adapted from Kaur et al. (2008).

The association of orexin neuron activity with systemic and behavioral variables relevant to food seeking behavior, and the broad distribution of orexinergic efferents within the central nervous system, makes this population of cells an intriguing candidate to mediate the effects of restricted feeding schedules on food anticipatory behavior. The results of ablation studies indicates that orexin neurons do play a role, but not as a driving oscillators responsible for timing anticipatory behavior. An early study using ibotenic acid to ablate lateral hypothalamic neurons nonspecifically in rats reported that while total daily activity was reduced, food anticipatory activity was not eliminated (Mistlberger and Rusak, 1988). Similar results were obtained using an orexin2-saporin toxin to ablate neurons expressing orexin2 receptors, which include neurons producing orexin (Mistlberger et al., 2003). A more selective genetic ablation of orexin neurons occurs developmentally in orexin/ataxin-3 mice. This is associated with behavioral state instability similar to narcolepsy, loss of the increase in consolidated wakefulness that occurs during acute food deprivation, and attenuation of food anticipatory activity (Hara et al., 2001b; Akiyama et al., 2004; Mieda et al., 2004). Orexin ligand knockout mice may also exhibit a reduction in food anticipatory activity compared to WT mice, but nonetheless exhibit a comparable peak of activity at the expected mealtime during a total food deprivation test (Kaur et al., 2008) (Figures 4D–F). Circadian timing of food anticipatory activity can thus be described as more accurate and thus more efficient in these mice. The same study reported no deficit in the expression of the food anticipatory rise of body temperature (Figures 4A–C), and a second study, using a video-based activity recording system detected no reduction in food anticipatory activity in orexin ligand KO mice (Gunapala et al., 2011). These results indicate that the effects of orexin deficiency on food anticipatory activity depend on the variable measured. Orexin neurons appear to play an important role regulating behavioral state stability and promoting wake consolidation during caloric restriction, but orexin and co-localized neuromodulators do not function as FEOs critical for timing food anticipatory activity or body temperature. Orexin neurons do interact with neural substrates mediating reward (Narita et al., 2006; Choi et al., 2010) and are activated in anticipation of reward (Harris et al., 2005), but a role in anticipation of palatable foods or other rewards provided on a circadian schedule has not yet been evaluated.

A portion of the orexinergic neuron population overlaps with the dorsomedial hypothalamus, which contains Mc3 receptors and neurons involved in regulating autonomic and neuroendocrine functions. Some neurons in this area exhibit daily rhythms of clock gene and immediate-early expression in rats and mice anticipating a daily meal (Gooley et al., 2006; Mieda et al., 2006; Verwey and Amir, 2009). Cell body specific lesions of this area may attenuate food anticipatory activity (Gooley et al., 2006; Acosta-Galvan et al., 2011; Landry et al., 2011), but complete removal of the area by radiofrequency ablation does not (Landry et al., 2006, 2007; Moriya et al., 2009). Also, clock gene oscillations in the dorsomedial hypothalamus are not induced in rats anticipating a daily palatable meal without caloric restriction, but are induced in rats maintained on a restricted feeding schedule with a random daily mealtime, which does not entrain anticipatory rhythms (Angeles-Castellanos et al., 2008; Verwey et al., 2009). These results indicate that circadian oscillators in the dorsomedial hypothalamus are neither necessary nor sufficient for the induction of food anticipatory activity rhythms. Dorsomedial hypothalamic neurons are responsive to feeding related signals, and contain neurons projecting to the light-entrainable circadian clock in the SCN that may inhibit its output during the daily rest phase, thereby promoting the expression of food anticipatory activity (Acosta-Galvan et al., 2011; Landry et al., 2011).

## Summary and conclusions

Nocturnal animals normally work for food and eat at night. Scheduled feeding can markedly alter the timing of behavioral arousal and locomotor activity, resulting in concentrated periods of waking and food seeking behavior that anticipate expected meals, even if these occur during the usual rest phase of the circadian cycle. Similar changes have been reported in response to time-limited opportunities to obtain other rewards, including water, sex, and addictive drugs (Webb et al., 2009b). These behavioral changes are associated with shifting of circadian oscillators in the brain and in the periphery. However, the endocrine and neural substrates of entrainment of activity rhythms by food (and other rewards) remain to be clarified. Metabolic hormones and the hypothalamic circuits upon which they converge appear to play modulatory roles, increasing or decreasing the amplitude or duration of food anticipatory rhythms without being required for induction or persistence of these rhythms. These results, in combination with other lesion studies (Mistlberger, 1994; Davidson, 2009), suggest that FEOs critical for behavioral rhythms are either located outside of the hypothalamus, or are widely distributed and substantially redundant within the hypothalamus. Circadian oscillators reset by daily feeding schedules are also found in other brain regions, including mesocorticolimbic and dorsal striatal circuits that participate in reward processing and motivation for natural and drug reward (Verwey and Amir, 2009; Webb et al., 2009b). There is no direct evidence available yet that reward circuits are necessary or sufficient for entraining or driving food anticipatory circadian rhythms, but this remains a testable hypothesis.

Circadian clock adjustments induced by food intake are presumed to be adaptive, by coordinating rest-activity cycles with food availability, and metabolic functions with food ingestion. However, the sensitivity of circadian oscillators to food or other rewards could be maladaptive in at least two ways. First, entrainment to powerful rewards (e.g., palatable food, addictive drugs) could induce daily temporal windows of vulnerability to excessive reward seeking behavior. Second, feeding stimulated at irregular mealtimes could result in disrupted timing of metabolic processes, with negative consequences for metabolic functioning and health (Arble et al., 2010; Golombek et al., 2013). To the extent that stress alters food intake, food sensitive circadian clocks may therefor represent a pathway from stress to disease. This suggests a research agenda exploring effects of stress on temporal parameters of food intake, and associated changes in phase relationships among circadian oscillators that control behavior and physiology. This idea remains to be evaluated experimentally, but would benefit from progress in specifying the neurobiological mechanisms that mediate the effects of food intake on circadian rhythms.

### Conflict of interest statement

The authors declare that the research was conducted in the absence of any commercial or financial relationships that could be construed as a potential conflict of interest.
